# Proteomic analysis of urinary extracellular vesicles from high Gleason score prostate cancer

**DOI:** 10.1038/srep42961

**Published:** 2017-02-17

**Authors:** Kazutoshi Fujita, Hideaki Kume, Kyosuke Matsuzaki, Atsunari Kawashima, Takeshi Ujike, Akira Nagahara, Motohide Uemura, Yasushi Miyagawa, Takeshi Tomonaga, Norio Nonomura

**Affiliations:** 1 Department of Urology, Osaka University Graduate School of Medicine, Osaka, Japan; 2 Laboratory of Proteome Research, National Institute of Biomedical Innovation, Health and Nutrition, Osaka, Japan

## Abstract

Extracellular vesicles (EVs) are microvesicles secreted from various cell types. We aimed to discover a new biomarker for high Gleason score (GS) prostate cancer (PCa) in urinary EVs via quantitative proteomics. EVs were isolated from urine after massage from 18 men (negative biopsy [n = 6], GS 6 PCa [n = 6], or GS 8–9 PCa [n = 6]). EV proteins were labeled with iTRAQ and analyzed by LC-MS/MS. We identified 4710 proteins and quantified 3528 proteins in the urinary EVs. Eleven proteins increased in patients with PCa compared to those with negative biopsy (ratio >1.5, p-value < 0.05). Eleven proteins were chosen for further analysis and verified in 29 independent urine samples (negative [n = 11], PCa [n = 18]) using selected reaction monitoring/multiple reaction monitoring. Among these candidate markers, fatty acid binding protein 5 (FABP5) was higher in the cancer group than in the negative group (p-value = 0.009) and was significantly associated with GS (p-value for trend = 0.011). Granulin, AMBP, CHMP4A, and CHMP4C were also higher in men with high GS prostate cancer (p-value < 0.05). FABP5 in urinary EVs could be a potential biomarker of high GS PCa.

Elevation of the prostate-specific antigen (PSA) level and/or an abnormal digital rectal examination (DRE) leads to prostate needle biopsy to diagnose prostate cancer. However, up to 40% of patients newly diagnosed with prostate cancer were categorized as a low-risk group^1^. These patients with low-risk prostate cancer had a very limited possibility of disease progression and did not require definitive therapy. It is also well recognized that PSA lacks specificity and sensitivity, leading to unnecessary prostate biopsy. The Gleason classification is an established prognostic indicator that is scored based on the histologic pattern of the arrangement of cancer cells. Needle biopsy Gleason grade is routinely used for guiding patient management decisions^2^. It is controversial whether GS6 prostate cancer should be labeled as cancer because patients with GS6 prostate cancer have a similar prognosis with or without treatment^3^. The PSA test cannot differentiate between aggressive and benign prostate disease and leads to overdiagnosis and unnecessary biopsies^2^, and these issues led the U. S. Preventive Services Task Force to recommend against PSA-based screening for prostate cancer. Therefore, the development of a new marker for the diagnosis of high GS prostate cancer is necessary^3^,^4^,^5^,^6^.

Urine is a promising source of new biomarkers of prostate cancer, and several urinary markers have been reported, such as PCA3 and the TMPRSS2-fusion gene^7^,^8^,^9^. Recently, urine collected after prostate massage was reported to contain extracellular vesicles (EVs) that are secreted from prostate cancer cells^10^,^11^.

EVs, such as exosomes and microvesicles, are small vesicles (30–1000 nm in diameter) secreted from various types of cells and exist in bodily fluids such as blood, urine, ascites, and saliva. EVs contain microRNAs, proteins, and mRNAs and play a role in intercellular communications via the mechanisms of exocytosis and endocytosis^12^,^13^. EVs enhance the metastasis of cancer by transmitting their contents to cells such as endothelial cells and stromal cells in distant locations or tumor microenvironments. EVs are characterized by the presence of tetraspanins (CD9, CD63, and CD81) on their membranes and membrane fusion proteins such as Rab. Because microRNAs, proteins, and mRNAs in EVs may reflect the originating prostate cancer cells^12^,^13^, EVs could be potential sources of the discovery of new biomarkers for prostate cancer^14^,^15^,^16^,^17^. Recently, microRNAs in urinary EVs were reported to be biomarkers of prostate cancer^18^,^19^.

Recent advances in quantitative proteomic technology have enabled the large-scale quantitation and validation of biomarker candidates. Improvements in LC-MS technology have led to an increase in the number of proteins identified, and stable isotopic labelling methods using isobaric tags for relative and absolute quantitation (iTRAQ) have enabled the quantitative analysis of multiple samples simultaneously^20^. Selected reaction monitoring/multiple reaction monitoring (SRM/MRM) can measure the multiple proteins at high sensitivity and throughput without antibodies^21^. Cancer-cell-derived EVs can be measured by two types of antibodies for CD9 and the biomarker protein in a high-throughput manner^22^. In this study, we performed quantitative proteomic analysis of EV proteins from urine collected after prostate massage to discover potential biomarker candidates for the diagnosis of high GS prostate cancer and then verified the candidate proteins.

## Results

### Confirmation of EVs

**Figure 1:**
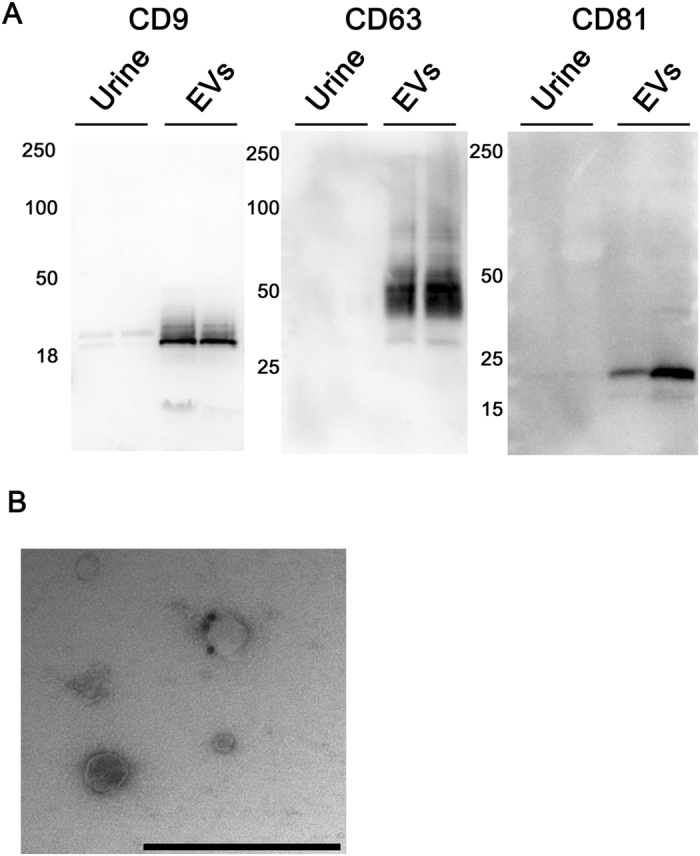
Extracellular vesicles (EVs) isolated from urine. (**A**) Western blotting showed the expression of specific proteins (CD9, CD63, and CD81) in urinary EVs. (**B**) Electron microscopy shows urinary exosomes immunolabeled with anti-CD9 and attached to 20-nm protein gold nanoparticles. Bar indicates 500 nm.

Urinary EVs collected after prostate massage were extracted by ultracentrifugation. Proteins extracted from EVs were enriched with CD9, CD63 and CD81 proteins, which are markers of EVs, compared with unprocessed urinary proteins (Fig. 1A). EVs labeled with anti-CD9 antibody conjugated with Au colloids were also confirmed by electron microscopy (Fig. 1B).

### iTRAQ Analysis

**Figure 2:**
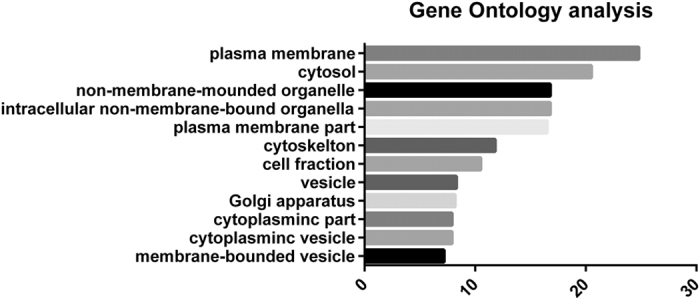
Gene ontology annotation of identified proteins from extracellular vesicles in urine collected after prostate massage. Cellular components.

We performed shotgun proteomics of EVs in urine collected after prostate massage to identify potential biomarker candidates for GS prostate cancer. In total, 18 samples (negative: n = 6; GS 6: n = 6; GS 8–9: n = 6) were labeled with iTRAQ (isobaric tag for relative and absolute quantitation) and analyzed with liquid chromatography-tandem mass spectrometry (LC-MS/MS). Patient characteristics are summarized in Table 1. A total of 4710 unique proteins were identified, from which 3528 unique proteins were quantified using 6 iTRAQ analysis sets. Gene ontology (GO) cellular component analysis showed that the most abundant proteins that could be derived from EV proteins were plasma membrane proteins (24.8%) (Fig. 2). Eleven proteins increased in patients with PCa compared to those with negative biopsy (ratio >1.5, p-value < 0.05). The iTRAQ results of these 11 biomarker candidates are summarized in Table 2, and the iTRAQ results of all quantified proteins are summarized in Supplemental Table.

### Verification of Biomarker Candidates by SRM/MRM

**Figure 3:**
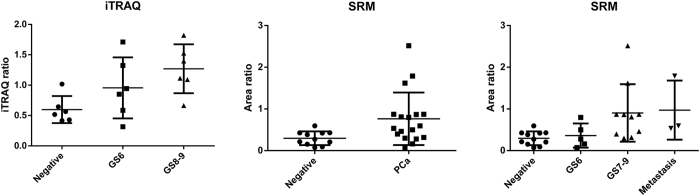
iTRAQ and SRM/MRM data for FABP5 in urinary extracellular vesicles.

**Figure 4:**
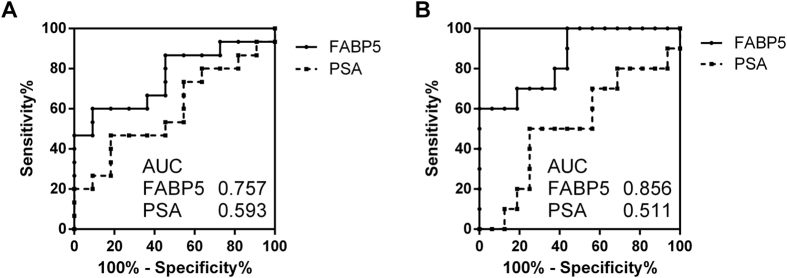
Receiver-operator characteristic (ROC) curve for the detection of prostate cancers (**A**) and cancers with a Gleason score ≥ 7 (**B**) on biopsy by FABP5 levels in EVs from the urine after DRE (solid line) and serum PSA (dashed line).

We selected 11 proteins for verification with SRM/MRM. The selection criterion was an iTRAQ ratio of cancer/negative biopsy of >1.5 (p-value < 0.05) (Table 2). Twenty-nine urine samples collected after prostate massage (11 urine samples from men with negative biopsy and 18 urine samples from men with prostate cancer [5 from men with GS 6, 7 from men with GS 7, and 6 from men with GS 8–9]) were used for verification as the independent cohort. Three patients with GS 8–9 had distant metastasis of prostate cancer. Among the 11 candidate proteins, only fatty acid binding protein 5 (FABP5) was significantly overexpressed in men with prostate cancer compared with men with negative biopsy (p-value = 0.009). Moreover, FABP5 was significantly associated with GS (p-value for trend = 0.011) (Fig. 3). The receiver-operator characteristics (ROC) curve analysis showed that the area under the curve (AUC) for the prediction of prostate cancer with GS ≥ 6 by FABP5 was 0.757 (95% confidence interval [CI] 0.570–0.944, p-value = 0.027), whereas the AUC value was 0.593 (95% CI 0.372–0.815, p-value = 0.42) for prediction by serum PSA (Fig. 4A). Granulin and alpha-1-microglobulin/bikunin precursor (AMBP) were also significantly associated with GS (p-value for trend = 0.011 and 0.014, respectively), whereas neither protein was significantly overexpressed in men with prostate cancer compared with men with negative biopsy (p-value = 0.90 and 0.59, respectively) (Supplemental Fig. 1). Charged multivesicular body protein 4a (CHMP4A), charged multivesicular body protein 4c (CHMP4C), and charged multivesicular body protein 2b (CHMP2B) were overexpressed in men with GS 8–9 (p-value = 0.016, 0.042, and 0.053, respectively), whereas CHMP4A, CHMP4C, and CHMP2B were not significantly overexpressed in men with prostate cancer compared to men with negative biopsy (p-value = 0.83, 0.70, and 0.67, respectively) (Supplemental Fig. 2). Next, we determined whether FABP5 in urinary EVs after massage could predict prostate cancers with GS of 7 or more, which requires definitive treatment, in the cohort without metastasis (n = 26). Univariate logistic analysis showed that FABP5 was significantly associated with prostate cancers with GS 7 or more (p-value < 0.001). Even after adjusting for age, PSA, and PSA density, FABP5 was significantly associated with prostate cancer with GS 7 or more (p-value = 0.003) (Table 3). The ROC curve analysis showed that the AUC for the prediction of GS ≥ 7 by FABP5 was 0.856 (95% CI 0.708–1.00, p-value = 0.002), whereas the AUC value for prediction by serum PSA was 0.511 (95% CI 0.280–0.757, p-value = 0.87) (Fig. 4B). The sensitivity and specificity of FABP5 at the best cutoff value were 60.0% and 100%, respectively.

### FABP5 Expression in Prostate Cancer

**Figure 5:**
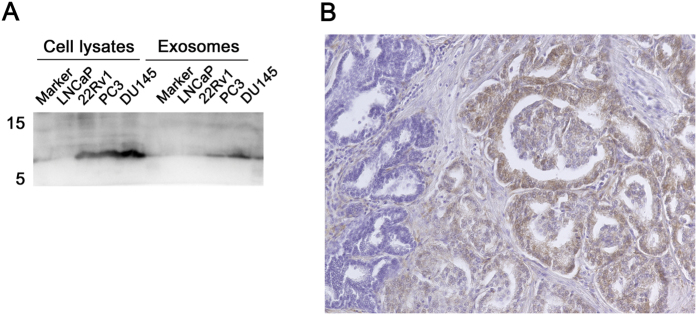
FABP5 expression in prostate cancer. (**A**) Western blot analysis of FABP5 expressions in cell lysates of prostate cancer cell lines and extracellular vesicles in cell culture media. (**B**) Immunohistochemical analysis of FABP5 in prostatectomy specimens.

We studied FABP5 expression in prostate cancer cell lines and prostate cancer tissues. Western blot analysis showed that FABP5 was expressed in EVs from the supernatants of PC3 and DU145 prostate cancer cell lines (Fig. 5A). Immunohistochemical analysis of prostatectomy specimens from the patients with prostate cancer showed that prostate cancer cells with Gleason pattern 4 were stained with anti-FABP5 antibody in contrast to the negative normal epithelium (Fig. 5B). These results suggested that the prostate cancer cells could be the origin of EVs with FABP5.

## Discussion

The development of new biomarkers for high-risk prostate cancer is continuing by many researchers using various methods. In the prostatectomy specimens, expressions of proteins and mRNA were used to predict the prognosis of prostate cancer patients. Immunohistochemical analysis showed that the expression of annexin II in prostatectomy specimens was associated with the Gleason score^3^. The expression of pre-B cell leukemia homeobox transcription factor was associated with cancer-specific survival in patients who underwent prostatectomy^5^. Quantitative phase imaging with prostatectomy specimens could predict prostate cancer recurrence^4^. In serum, the monocyte fraction was associated with the Gleason score^6^. Plasma prostate-specific EV concentrations, which were measured by an ELISA kit for prostate-specific membrane antigen, differed in patients with benign prostatic hypertrophy and low-, intermediate-, and high-risk prostate cancer^17^. In the urine, glycosylated PSA was associated with the Gleason score^8^.”

EVs were first reported in 1983^23^, and with recent advances in technology, EVs are now emerging as a resource for biomarker discovery in several types of cancer. In prostate cancer, several miRNAs (such as miR-21 and miR-107) and mRNA (AGR2 splice variant, PCA3, and TMPRSS-ERG) have been reported to be biomarkers in urinary EVs^11^.

The results of proteomic analysis of EVs in urine collected after prostate massage from men with prostate cancer were previously reported, but the number of detected proteins was up to 900 proteins^24^. Overbye *et al*. also reported the proteomic analysis of EV in urine collected without prostate massage^25^. They compared the urinary EVs from normal volunteers and men with prostate cancer and identified 1644 proteins in urinary EVs, but their urine samples were collected without prostate massage, which might have resulted in the detection of different urinary markers for prostate cancer. In the present study, we quantified 3528 proteins by deep proteomic analysis, and, to our knowledge, this is the largest number of EV proteins ever detected in urine from patients with prostate cancer. However, only FABP5 was significantly overexpressed in EVs in men with prostate cancer compared to men with negative biopsy in the verification cohort. This might be due to the small number of men in the verification cohort or that the limit of biomarker discovery in urinary EVs was reached with the current technique.

FABP5, also called C-FABP5 and E-FABP5, is a lipid binding protein that is found in epidermal cells^26^. FABP5s play roles in fatty acid uptake, intracellular transport, and metabolism. FABP5 may also play a role in stimulating tumor progression by transferring ligands to nuclear PPARβ/γ^27^. FABP5 promotes cell proliferation and invasion in cholangiocarcinoma^28^. Proteomic analysis of prostate cancer and benign tissue showed that FABP5 was upregulated in prostate cancer tissues^29^. Another proteomic study of surgical specimens of prostate cancer showed that tissues from lymph node metastasis had increased expression of FABP5 compared with localized prostate cancer tissues^30^. Interestingly, patients with lymph node metastasis had higher levels of serum FABP5, as measured by ELISA^30^. FABP5 promotes the proliferation of endothelial cells^31^. The present study showed the high expression of FABP5 in EVs from patients with high GS prostate cancer. FABP5 in EVs from high GS prostate cancer may affect the endothelial cells in tumor microenvironments or in distant areas and promote the metastasis of prostate cancer.

GRN, also known as granulin-epithelial precursor, is the pluripotent growth factor regulating inflammation and tumorigenesis. GRN is reported to be overexpressed in high-grade prostatic intraepithelial neoplasia and invasive prostate cancer compared with benign prostate epithelium^32^ and to stimulate the migration, invasion, and proliferation of prostate cancer^33^. GRN-overexpressed hepatocellular carcinoma (HCC) cells had stem cell-like properties, and patients with GRN-overexpressed HCC had a poor prognosis^34^. GRN was also secreted from the bone marrow cells and supported the stromal activation and tumor growth in mouse breast cancer models^35^. GRN-secreting monocytes play a key role in the liver metastasis of pancreatic ductal adenocarcinoma^36^. AMBP plays a role in the regulation of inflammation and was reported to be a biomarker of prostate cancer and bladder cancer by proteomic analysis of urine^29^,^37^. High levels of serum AMBP predict the poor response of gastric cancer patients treated with chemotherapy^38^. CHMP4A, CHMP4C, and CHMP2B belong to the chromatin-modifying protein/charged multivesicular body protein family and are components of ESCRT-III (endosomal sorting complex required for transport III) involved in the formation of endocytic multivesicular bodies^39^. *CHMP4A* expression is associated with the recurrent ovarian cancer^40^, and CHMP4C plays roles in radiation resistance in non-small cell lung cancer^41^. GRN, AMBP, CHMP4A, CHMP4C, and CHMP2 may play important roles in aggressive prostate cancer and may be potential targets of treatment.

This study has several limitations. First, the sample size is small, and the issue of multiple tests for analyzing the iTRAQ results exists. Further large-scale studies are warranted. Second, there are several hurdles to applying these findings to clinical settings. The development of simpler and easier measurements is mandatory. The isolation of EVs by multiple ultracentrifugation is not suitable in the clinical setting because the isolation process takes more than 4 hours. Recently, several one-step isolation kits for EVs have been developed^42^ whose use might circumvent these issues. Proteins located on the membrane of EVs can be measured by these new methods in a high-throughput manner. GO showed that FABP5 is located in the cytoplasm, and FABP5 could also be located inside the EVs. To measure the protein inside the EVs, ExoScreen, which uses two types of antibodies against the proteins located on the membrane of EVs, could not be applied^22^. A patent entitled “Biomarker compositions and methods (WO 2014082083 A1)” describes a technique to detect cancer-associated EVs in bodily fluids by staining lipid layers of EVs, and various cancer-associated antigens including FABP5 were described as potential microvesicle-associated antigens. This method could be a possible technique to detect EVs without ultracentrifugation in clinical settings. Recently, mass-spectrometry analysis has been brought to clinical tests in the hospital. After isolation of the EVs from urine with the one-step kit, biomarkers could be quantified by mass-spectrometry. Further development of mass spectrometry technology might solve these problems. Third, the biological meanings of high amounts of FABP5 in urinary EVs from patients with high GS should be studied.

In conclusion, we applied the proteomic analysis to discover biomarkers in EVs in urine collected after prostate massage. FABP5 in urinary EVs could be a potential biomarker of high GS prostate cancer. Additional large-scale studies are warranted to confirm this finding.

## Material and Methods

### Sample Collection

Urine samples were collected in Osaka University Hospital. Approval was obtained from the Osaka University Graduate School of Medicine Institutional Review Board before initiating the study, and all patients gave written informed consent. All methods were performed in accordance with the relevant guidelines and regulations. Initial voided urine (20–100 mL) was prospectively collected from patients before prostate biopsy, immediately following DRE. In all cases, the DRE was performed as 3 finger strokes per prostate lobe. Voided urine samples were kept at 4 °C for up to 6 h prior to preparing aliquots of the urine samples and then centrifuged at 2000 × *g* for 30 min. After removing the pellets, supernatants of urine were stored at −80 °C until analysis.

### Patient Classification

We determined Gleason grades from the reports of the pathologists in Osaka University Hospital based on classifications decided at the 2005 ISUP Consensus Conference. The “negative” group comprised the patients who received prostate biopsy due to elevated PSA levels and were diagnosed pathologically as negative. The “GS6”, “GS7”, and “GS8–9” groups included the patients diagnosed pathologically as having prostate cancer with GS 6, GS7, and GS 8 or 9, respectively. The “PCa” group was defined as patients with prostate cancer of any GS. In the verification cohort, low-risk prostate cancer was defined as GS6 prostate cancer, and high-risk prostate cancer was defined as GS7, GS8, or GS9 prostate cancer. In the discovery cohort for iTRAQ analysis, 18 samples (negative: n = 6; GS 6: n = 6; GS 8–9: n = 6) were analyzed. In the verification cohort for SRM/MRM analysis, 29 samples (negative: n = 11, the low risk PCa: n = 5, the high risk PCa: n = 13) were analyzed.

### EV Isolation

Urine samples were centrifuged at 17,000 × *g* for 30 min to remove debris and then ultracentrifuged at 100,000 × *g* for 90 min. The supernatant was removed, and the pellets were washed with PBS and ultracentrifuged at 100,000 × *g* for 90 min. To remove the Tamm-Horsfall protein, the pellets were incubated with DTT for 30 min and ultracentrifuged at 100,000 × *g* for 90 min. The pellets containing EVs were suspended in 20 μL of PBS and frozen at −80 °C. EVs were solubilized with MPEX PTS reagent solution (GL Science, Tokyo, Japan) at 95 °C for 5 min followed by sonication for 5 min using a Bioruptor sonicator (Cosmo Bio, Tokyo, Japan). After centrifugation at 100,000 × *g* for 30 min at 4 °C, the supernatant was obtained as an EV fraction extract and quantified using Pierce Microplate BCA Protein Assay Kit-Reducing Agent Compatible (Thermo Scientific, Waltham, MA, USA).

### SDS-PAGE and Western Blot Analysis

Protein samples were electrophoretically separated on a precast Novex 4–12% Bis-Tris NuPAGE gel using MOPS running buffer (Invitrogen, Carlsbad, CA) according to the manufacturer’s instructions. The gels were stained with SYPRO Ruby Protein Gel Stain to confirm the sample quality. Other gels were transferred to polyvinylidene difluoride membranes (Millipore, Bedford, MA). Membranes were immunoblotted with mouse monoclonal anti-human CD9 antibodies (12A12; Shionogi, Osaka, Japan) or rabbit monoclonal anti-human FABP5 antibody (D1AA7T; Cell Signaling Technology, Danvers, MA, USA) followed by horseradish peroxidase-conjugated secondary antibodies and developed with a Super Signal West Dura Extended Duration Substrate kit (Thermo Fisher Scientific).

### Transmission Electron Microscopy (TEM)

Samples of EVs (10 μg) were adsorbed onto a formvar/carbon-coated nickel grid for 1 h. EVs were fixed with 2% paraformaldehyde and then reacted with the first antibody (anti-CD9 antibody). Immunoreactive EVs were visualized with the second antibody preabsorbed with 20-nm gold particles (anti-mouse IgG antibody). The samples were negatively stained with 2% aqueous uranyl acetate for 15 min and observed with a JEM-1400Plus transmission electron microscope (JEOL Ltd., Tokyo, Japan).

### iTRAQ Labeling

iTRAQ labeling was performed as previously described^21^. The reference pool was arranged by mixing an equal amount (40 μg) of 18 EV protein extracts. The EV fraction extract (15 μg) for iTRAQ labeling was reduced with 5 mM DTT for 30 min and then alkylated with 50 mM iodoacetamide for 30 min at room temperature after the addition of bovine serum albumin as the internal standard. The sample was digested with 1% trypsin overnight at 37 °C. The phase-transfer surfactants method was used to remove the surfactants^21^. The tryptic digest sample was desalted using C18 stage tips and then suspended in 30 μL of iTRAQ dissolution buffer and labeled with 4-plex iTRAQ reagents (Applied Biosystems, Foster City, CA) for 1 hr at room temperature. The tryptic digests of the reference pool, negative group (n = 6), prostate cancer with GS 6 (GS 6 group, n = 6), and prostate cancer with GS 8 or 9 (GS 8–9 group, n = 6)) were labeled with 4-plex iTRAQ reagents 114, 115, 116, and 117 (Table 1). The labeled samples were then pooled and desalted using C18 stage tips. iTRAQ-labeled peptides were divided into 58 fractions using an HPLC system (Shimadzu Prominence UFLC). We monitored the concentrations of these fractions by UV spectroscopy and then combined low-concentration fractions, which resulted in 17 fractions. The 115:114, 116:114, and 117:114 ratios indicated the relative abundance of proteins in the negative group, GS 6 group, and GS 8–9 group, respectively, relative to the common reference pool.

### LC-MS/MS

The fractionated peptides were analyzed by nano-LC-MS/MS using LTQ-Orbitrap LTQ Velos Elite (Thermo Fisher Scientific, Bremen, Germany) with a nano-LC interface (AMR, Tokyo, Japan), Paradigm MS2 (Michrom Bioresources, Auburn, CA), and HTC PAL autosampler (CTC Analytics, Zwingen, Switzerland). Each fraction was injected into a trap column (0.3 × 5 mm, L-column ODS; Chemicals Evaluation and Research Institute [CERI], Tokyo, Japan) and separated on an analytical column (0.1 × 200 mm in-house developed Tip Column packed with L-column2 C18 particles; CERI). MS/MS conditions used for iTRAQ analysis were set as follows: the 12 most intense precursor ions were selected for the MS/MS scans, which were performed using higher energy collision-induced dissociation (HCD) (scan range 350–1500 *m/z*, with 30K FWHM resolution at 400 *m/z*). The normalized collision energy value was set to 35%. The MS/MS isolation width was set to 1.5 Da. A dynamic exclusion option was implemented with a repeat count of 1 and exclusion duration of 60 s. The automated gain control (AGC) values were set to 1.00e + 06 for full MS and 5.00e + 04 for HCD MS/MS. A lock mass ion (*m/z* = 391.28 and 445.12) was used for internal calibration.

### Identification and Quantification of Proteins

Raw data were examined using Proteome Discoverer ver.1.4 (Thermo Fisher Scientific, Bremen, Germany) with Mascot v2.4 (Matrix Science, London, UK) against Uniprot (UP Human without isoform, 14.3 release version), following LC-MS/MS analysis. High-confidence peptide identification was obtained by setting a target FDR threshold of <1.0% at the peptide level. A minimum of two peptides meeting the criteria were required for protein identification. Protein quantification was performed using Proteome Discoverer ver.1.3. iTRAQ ratios, 115:114, 116:114, and 117:114, of each experiment were normalized according to the iTRAQ ratios of CD9 signals to normalize for deviations in EV collection from urine. Protein quantification was performed using only unique peptide. The quantitation values were calculated on the basis of the intensity of the iTRAQ reporter ions in the HCD scans using Proteome Discoverer.

### SRM/MRM Analysis

We used SRM/MRM to confirm and further verify the biomarker candidates obtained from iTRAQ. SRM/MRM was performed as previously described^10^,^11^. A total of 27 EVs were isolated from urine samples from men with negative biopsy (n = 11), men with low-risk prostate cancer (n = 5), and men with high-risk prostate cancer (n = 13) (Table 1). Stable synthetic isotope-labeled peptides (SI peptides) with a C-terminal 15N- and 13C-labeled arginine or lysine residue (isotopic purity >99%) were purchased from Greiner Bio One (Frickenhausen, Germany) (crude purity). The peptide sequence was selected from the unique peptide sequences identified in the iTRAQ experiments. Among the 17 proteins with iTRAQ ratios of cancer/negative biopsy of >1.5 (p-value < 0.05), six proteins were excluded because appropriate SI peptides could not be designed. The correction for multiple tests was not performed. The SI peptide mixture was analyzed by the above-mentioned LCs-MS/MS method using LTQ Orbitrap-XL to acquire MS data. A preliminary SRM/MRM transition list for SI peptides was created from the MS data acquired using Pinpoint ver.1.0 (Thermo Fisher Scientific, Bremen, Germany). An EV fraction extract (2 μg) prepared from validation sets of urine samples was reduced with DTT, alkylated with iodoacetamide, and then digested as described above for quantitation using SRM/MRM. The digested peptide was analyzed using the above-described optimal-timed SRM/MRM method with a TSQ-Vantage triple quadruple mass spectrometer (Thermo Fisher Scientific, Bremen, Germany). The parameters used for SRM/MRM analysis were set as follows; scan width of 0.002 *m/z*, Q1 and Q3 resolution of 0.7 fwhm, cycle time of 2.5 s, and a gas pressure of 1.8 mTorr. Collision energy (CE) was optimized for each transition by performing a test run of the SI-peptide mixture. The SI peptide mixture was added to the trypsin-digested sample, and the area ratio of the endogenous peptide to the SI peptide was calculated using the transition peak area measured with Pinpoint software. Because the SI peptides used were of crude purity, the quantitated values of the endogenous peptide are relative amounts between samples and not absolute amounts. The amount of each SI peptide added to each sample was adjusted to be similar to the endogenous peptide estimated by the peak area obtained from preliminary SRM/MRM of the sample mixture. The average of these ratios of more than two transitions was first calculated, and the average ratio of two technical replicates of an individual sample was then determined as the relative quantitative value of the target peptide. The signal-to-noise ratio was identified using Pinpoint software. CD9 proteins were also measured to normalize EVs in urine.

### Immunohistochemistry

Immunostaining was performed as previously reported^43^. Briefly, sections were deparaffinized using xylene and alcohol and incubated with 0.3% H_2_O_2_ to block endogenous peroxidase activity. Before immunostaining, the antigen was retrieved by immersing the sections in 10 mmol/L citrate buffer (pH 6.0) and placing them in steam above boiling water for 20 min. Immunohistochemical staining for FABP5 was carried out with anti-endoglin antibody (D1A7T, CST 1:600) using an EnVision + Detection System (DAKO, Glostrup, Denmark) according to the manufacturer’s instructions. Primary antibodies were incubated overnight at 4 °C and counterstained with hematoxylin.

### Data Analysis

Identified proteins were analyzed using a DAVID for GO cellular component and biological process annotation^44^. All statistical analyses were carried out using SPSS version 11.0.1 (SPSS, Chicago, IL, USA) and R version 2.13.0 with RcmdrPlugin. EZR package (Saitama Medical Center, Jichi Medical University), which is a graphical user interface for R (The R Foundation for Statistical Computing). More precisely, it is a modified version of R commander (version 1.6–3) designed to add statistical functions frequently used in biostatistics. Mann-Whitney tests were used to analyze the difference between two categories. Stepwise associations between GS and FABP5 levels were analyzed using the Jonckheere-Terpstra test. Significant factors predicting a GS ≥ 7 were identified by logistic regression analysis, and variables entered into the model were patient age and levels of PSA, PSA density, and FABP5.

## Additional Information

**How to cite this article**: Fujita, K. *et al*. Proteomic analysis of urinary extracellular vesicles from high Gleason score prostate cancer. *Sci. Rep.* **7**, 42961; doi: 10.1038/srep42961 (2017).

**Publisher's note:** Springer Nature remains neutral with regard to jurisdictional claims in published maps and institutional affiliations.

## Supplementary Material

Supplemental Table

Supplemental Figures

## Figures and Tables

**Table 1:** Patient characteristics in the discovery and verification cohorts.

Discovery cohort for iTRAQ quantitative proteomic analysis			
	Negative	GS6	GS8–9
N	6	6	6
Age	69 (49–73)	70 (58–79)	70 (63–83)
PSA	5.7 (4.6–11.7)	9.0 (4.1–126)	16.3 (6.8–311)
Gleason score		GS6: 6	GS8: 3
			GS9: 3
Verification cohort for SRM/MRM analysis			
	Negative	Low-risk PCa	High-risk PCa
N	11	5	13
Age	64 (59–71)	67 (61–75)	64 (53–74)
PSA	6.2 (4.4–21.7)	6.2 (2.9–10.6)	6.8 (4.3–3143)
Gleason score		GS6: 5	GS7: 7
			GS8: 3
			GS9: 3

**Table 2:** List of Biomarker candidates with iTRAQ ratio. PCa/Neg., average ratio of prostate cancer to negative. GS6/Neg., average ratio of GS6 prostate cancer to negative. GS8–9/Neg., average ratio of GS8–9 prostate cancer to negative.

Accession	Protein name	Gene name	PCa/Neg.	p-value	GS6/Neg.	p-value	GS8–9/Neg.	p-value
Q9Y6U3	Adseverin	SCIN	2.74	0.043	2.79	0.089	2.69	0.031
P02760	Protein AMBP	AMBP	2.72	0.041	2.69	0.090	2.76	0.035
Q01469	Fatty acid-binding protein, epidermal	FABP5	2.31	0.009	1.85	0.020	2.76	0.006
Q96CF2	Charged multivesicular body protein 4c	CHMP4C	2.19	0.040	2.46	0.041	1.93	0.066
Q9UQN3	Charged multivesicular body protein 2b	CHMP2B	2.14	0.028	2.42	0.019	1.86	0.080
Q9UQB8	Brain-specific angiogenesis inhibitor 1-associated protein 2	BAIAP2	2.07	0.031	2.22	0.040	1.91	0.047
P28799	Granulins	GRN	2.00	0.013	1.99	0.071	2.02	<0.001
Q9HCH5	Synaptotagmin-like protein 2	SYTL2	1.95	0.028	1.87	0.029	2.03	0.050
P27797	Calreticulin	CALR	1.87	0.024	1.89	0.035	1.85	0.053
Q9BY43	Charged multivesicular body protein 4a	CHMP4A	1.62	0.022	1.66	0.015	1.57	0.065
O43598	2′-deoxynucleoside 5′-phosphate N-hydrolase 1	DNPH1	1.51	0.034	1.42	0.048	1.59	0.051

**Table 3:** Stepwise logistic regression analysis of variables associated with Gleason score ≥ 7.

Variable included	Univariate			Multivariate		
	Odds ratio	95%CI	*P*-value	Odds ratio	95%CI	*P*-value
Age	0.90	0.74–1.07	0.27	0.95	0.74–1.19	0.67
PSA	1.04	0.95–1.20	0.33	0.75	0.38–1.12	0.17
PSA density	23.3	0.66–6.02 × 10^5^	0.11	2.84 × 10^6^	0.22–8.55 × 10^16^	0.08
FABP5	184	5.09–3.67 × 10^4^	<0.001	440	4.77–1.09 × 10^6^	0.003
