# Supplementary material for: Proteomic analysis of urinary extracellular vesicles from high Gleason score prostate cancer
Source: Sci Rep. 2017 Feb 17;7:42961. doi: 10.1038/srep42961 (PMC5314323; doi:10.1038/srep42961)

Supplementary informations

Proteomic analysis of urinary extracellular vesicles from high Gleason score prostate cancer

Kazutoshi Fujita[1*], Hideaki Kume[2], Kyosuke Matsuzaki[1], Atsunari Kawashima[1], Takeshi Ujike[1], Akira Nagahara[1], Motohide Uemura[1], Yasushi Miyagawa[1], Takeshi Tomonaga[2], Norio Nonomura[1]

[1]Department of Urology, Osaka University Graduate School of Medicine, Osaka, Japan

[2]Laboratory of Proteome Research, National Institute of Biomedical Innovation, Health and Nutrition, Osaka, Japan

Supplemental Figure 1. iTRAQ and SRM/MRM data for GRN and AMBP in urinary extracellular vesicles.

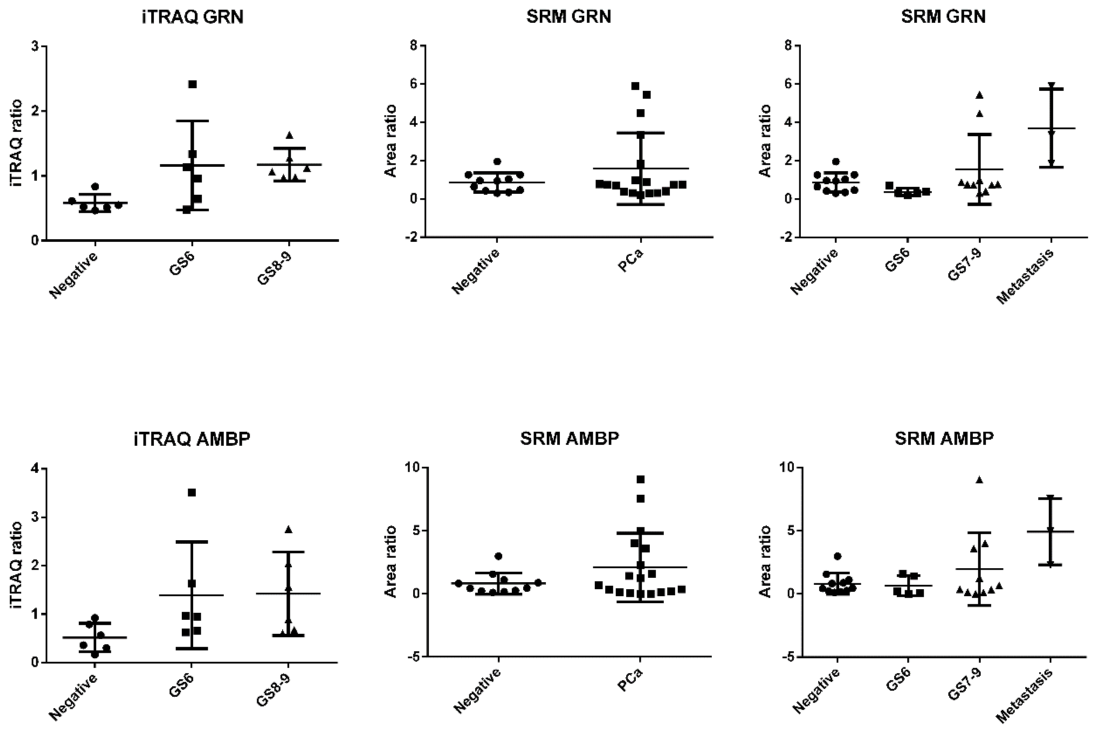

Supplemental Figure 2. iTRAQ and SRM/MRM data for CHMP4A, CHMP4B, and CHMP2B in urinary extracellular vesicles.

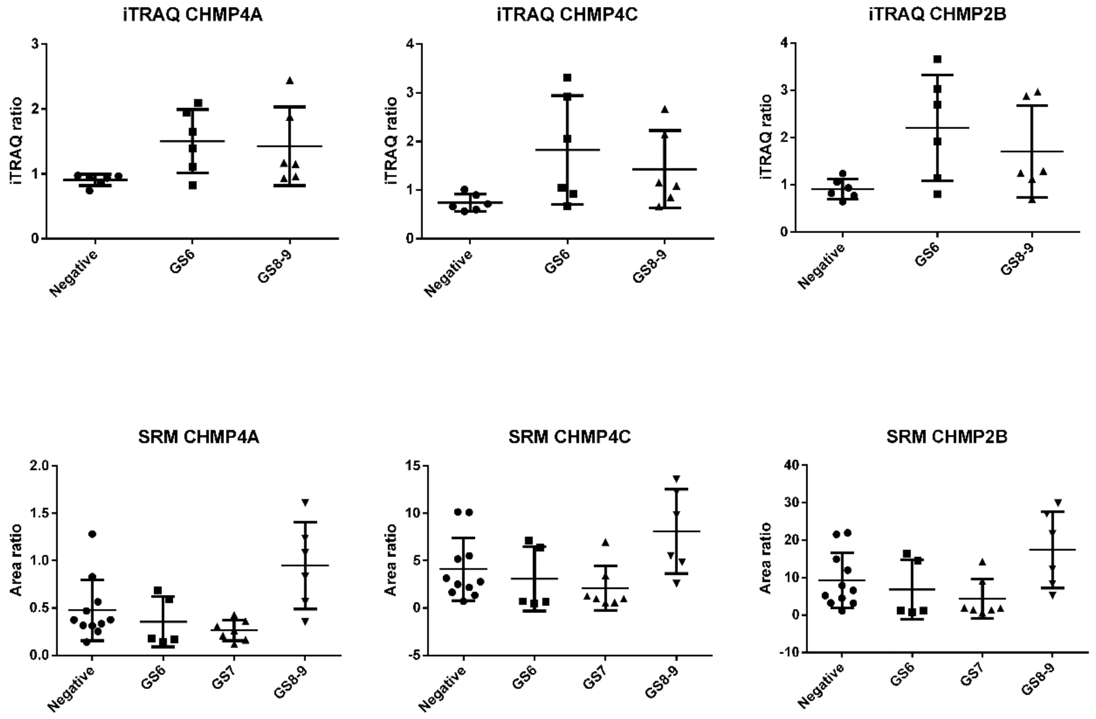

Supplement: Supplemental Figures [file srep42961-s2.pdf]
